# Targeting Cuproptosis and Ferroptosis via a ROS-Responsive Nanoplatform for Enhanced Synergistic Therapy Against Hepatocellular Carcinoma

**DOI:** 10.3390/antiox15060722

**Published:** 2026-06-05

**Authors:** Quan Zhu, Yangyang Zhang, Xinyi Zhu, Huijuan Zhang, Chuyu Xiao, Yingying Yang, Ting Huang, Jun Lu, Chang Liu, Chunjing Chen, Yueyuan Zhou, Tao Liu, Biyuan Liu, Fangguo Lu

**Affiliations:** 1 Department of Immunology, School of Medicine, Hunan University of Chinese Medicine, Changsha 410208, China; 004931@hnucm.edu.cn (Q.Z.); 202308010839@stu.hnucm.edu.cn (Y.Z.); 202308010330@stu.hnucm.edu.cn (H.Z.); 202408020133@stu.hnucm.edu.cn (C.X.); winlyl@stu.hnucm.edu.cn (Y.Y.); 202308010836@stu.hnucm.edu.cn (T.L.); 004205@hnucm.edu.cn (B.L.); 2 Department of Preventive Medicine, School of Medicine, Hunan University of Chinese Medicine, Changsha 410208, China; 202308020345@stu.hnucm.edu.cn (X.Z.); 004318@hnucm.edu.cn (J.L.); 3 Department of Pathology, School of Medicine, Hunan University of Chinese Medicine, Changsha 410208, China; 004929@hnucm.edu.cn; 4 Department of Histology and Embryology, School of Medicine, Hunan University of Chinese Medicine, Changsha 410208, China; liuchang004273@hnucm.edu.cn; 5 Department of Pathogenic Biology, School of Medicine, Hunan University of Chinese Medicine, Changsha 410208, China; 004787@hnucm.edu.cn (C.C.); zhouyueyuan1994@163.com (Y.Z.)

**Keywords:** ferroptosis, cuproptosis, elesclomol, liver cancer

## Abstract

Ferroptosis and cuproptosis are promising anti-tumor treatment strategies. Elesclomol (ES) is a kind of common cuproptosis inducer, and cisplatin (DDP) is a commonly used drug in liver cancer chemotherapy, which can induce cells to undergo ferroptosis. Both of these cell death processes require inducing cells to generate oxidative stress. Therefore, elesclomol and cisplatin may have a synergistic effect in anti-tumor treatment. Here, we designed an active oxygen-responsive nano-delivery system and conducted in vitro and in vivo to study the synergistic anti-liver cancer effect of elesclomol and cisplatin. Our data showed that the elesclomol nanoparticles can effectively inhibit the growth of liver cancer cells and showed extremely low organ toxicity. Elesclomol exhibited a synergistic effect with cisplatin in vitro, but the combined treatment of the two did not outperform single drug treatment in vivo. The reason might be that the nuclear factor erythroid 2-related factor 2 (Nrf2) protein in liver cancer cells is feedback-expressed, inhibiting the oxidative stress effects induced by elesclomol and cisplatin. Therefore, this study provides reference data for exploring the mechanism of elesclomol’s synergistic anti-liver cancer treatment with cisplatin and offers a feasible strategy for future precise liver cancer treatment and improving chemotherapy efficacy.

## 1. Introduction

Hepatocellular carcinoma (HCC) is a common malignant tumor in the digestive tract. In China, liver cancer is the fifth most common malignancy and the third most deadly, posing a severe threat to human health and survival. Most individuals show almost no symptoms in the early stages of HCC because of its sneaky development. When a diagnosis is obtained, the condition is frequently too advanced to be surgically cured. Targeted therapy and immunotherapy have made great strides in treating various tumor types in recent years [1,2,3], but there are still many issues that need to be resolved. For instance, almost all patients experience resistance to lenvatinib, a targeted treatment for HCC [4]. T cells can be successfully activated by anti-Programmed Death-Ligand 1 (PD-L1) antibodies to produce antitumor effects [5]. Nonetheless, a significant number of immature dendritic cells are found in the liver, which naturally creates a strong immunosuppressive environment [6]. Furthermore, in HCC patients, immunological markers such as PD-L1 show notable interindividual variability. Therefore, there is an urgent need for technological advancements in the treatment of advanced HCC.

Cuproptosis is a novel form of cell death proposed in 2022 [7]. Its primary mechanism involves Cu^2+^ entering cells and binding to acyl-containing components of the tricarboxylic acid cycle, causing aberrant acylated protein aggregation and Fe-S cluster protein loss. Cu^2+^ is then converted to Cu^+^, which precipitates inside mitochondria and eventually causes cell death. For Cu^2+^ to enter cells, a carrier is needed. The most used Cu^2+^ carrier at the moment is esclomol (ES). It enters cells where it dissociates to release Cu^2+^ intracellularly after binding with Cu^2+^ in a 1:1 molar ratio to create the copper complex esclomol-copper chloride (ESCu) [8]. Subsequently, Cu^2+^ interacts with ferredoxin 1 (*FDX1*) within mitochondria, where it is reduced to Cu^+^ and generates substantial reactive oxygen species. Cu^+^ induces protein toxicity stress, leading to cell death [9,10]. The liver serves as a copper-rich organ in the human body, thereby establishing a crucial prerequisite for employing copper-based therapies in the treatment of liver cancer. In vitro studies have demonstrated that ESCu effectively kills tumor cells in breast cancer, melanoma, and colorectal cancer, among others [11,12,13]. Preliminary studies have revealed that although ES exhibits extremely low cytotoxicity towards normal cells in vivo, its efficacy remains poor. This is mainly because it is difficult for ES that has not bonded to Cu^2+^ to have cytotoxic effects [14]. Moreover, ES is readily metabolized in the bloodstream, meaning only a very limited quantity enters tumor cells. However, it cannot be denied that ESCu induces copper-mediated death in tumor cells, representing an approach with significant potential for clinical application. Drug delivery systems based on nanotechnology possess the unique advantage of targeting specific tissues or cells, thereby creating a localized environment of high drug concentration and mitigating systemic side effects. Guo et al. found that no pathological damage or inflammatory response was seen in animal tissues, and that nanoscale ESCu particles correctly localized to tumor locations in mice. Both in vitro and in vivo, these particles successfully destroyed bladder cancer cells [15]. To enable precise identification of liver cancer cells within the body, we have designed a polymeric material with ROS-responsive capabilities for encapsulating ESCu. To encapsulate ESCu, we selected two types of nanoparticles. One of the particles has a surface covered with hyaluronic acid (HA), which is a ligand that can bind to CD44. CD44 is highly expressed on the surface of various malignant tumor cells, including hepatocellular carcinoma [16]. The other nanoparticle does not contain HA. Both of the two nanoparticles contain thioketal (TK) and possess reactive oxygen species response capability. Moreover, the two ESCu nanoparticles are presented in the form of liposomes. The liposomes load the ESCu complex within themselves or on the lipid bilayer, rather than being composed of pure ESCu particles. Therefore, the drug is delivered in the form of being attached to the liposomes.

Cisplatin (DDP) is a commonly used first-line chemotherapy drug for liver cancer. However, its therapeutic use is limited due to its poor response rate in HCC patients and several harmful side effects [17,18]. An important mechanism of DDP’s antitumor activity is the induction of ferroptosis in tumor cells [19]. However, several tumor cell types show large quantities of reducing products (most notably, aberrant activation of the nuclear factor erythroid 2-related factor 2 (Nrf2) signaling pathway), which reduce DDP-induced oxidative stress [20,21]. This represents a major mechanism underpinning tumor cell resistance to DDP. ESCu causes copper-dependent cell death by producing high intracellular reactive oxygen species (ROS) levels, which causes oxidative stress [22]. The feedback stimulation of Nrf2 signaling stimulates significant synthesis of reduced glutathione (GSH), allowing tumor cells to withstand DDP-induced ferroptosis [23,24,25]. In theory, this route might similarly protect against ESCu-induced copper-dependent cell death. Therefore, the purpose of this work is to elucidate the molecular processes by which ESCu kills hepatocellular carcinoma cells, as well as to explain potential ESCu resistance mechanisms in hepatocellular carcinoma cells. This provides critical prospective reference data for the future therapeutic use of ESCu and its equivalents in tumor therapy.

## 2. Materials and Methods

### 2.1. Cell Lines and Cell Culture

The cell lines used in this study included MHCC97-H cells, HCCLM3 cells, and LO2 cells; the accession numbers of the three cell lines are CVCL_4972, CVCL_6832, and CVCL_6926. Both MHCC97-H cells and HCCLM3 cells are humanized liver cancer cell lines, while LO2 cells are human normal liver cell lines. All three cell lines were purchased from Beina Biotechnology Co., Ltd., Xinyang, China. MHCC97-H and HCCLM3 cells were cultured in Dulbecco’s Modified Eagle Medium (DMEM) supplemented with 10% FBS at 37 °C with 5% CO_2_. LO2 cells were cultured in Roswell Park Memorial Institute 1640 Medium (RPMI-1640) supplemented with 10% Fetal Bovine Serum (FBS) at 37 °C and 5% CO_2_. When the cell density reached over 85%, the cells were digested with 0.25% trypsin, centrifuged at 800 rpm for 5 min, and then subcultured at a ratio of 1:2 for continued growth.

### 2.2. Main Reagents

The Cell Counting kit-8 reagent (CCK-8, Cat# A1867351) was purchased from AmBeed Company (Shanghai, China). Elesclomol (Cat# E126032), Cisplatin (Cat# C295225), and Copper Chloride (Cat# 7447-39-4) were purchased from Aladdin Company (Beijing, China). The Reactive Oxygen Species Fluorescence Probe 2′,7′-Dichlorodihydrofluorescein diacetate (DCFH-DA, Cat# CA1410) was purchased from Solvay Company (Brussels, Belgium). The Ferrous Ion Detection Kit (Cat# E1042-100) was purchased from Pulilai Gene Technology Co., Ltd. (Beijing, China). The Copper Ion Detection Kit (Cat# E010-1-1) was purchased from Nanjing Jiancheng Institute of Biotechnology Engineering (Nanjing, China). The lipid peroxide (LPO, Cat# BC5240) content detection kit was purchased from Solabio Company (Beijing, China). The rabbit-derived anti-FDX1 antibody (Cat# BS71332), rabbit-derived anti-Dihydrolipoamide S-acetyltransferase (DLAT) antibody (Cat# BS79900), rabbit-derived anti-Glutathione Peroxidase 4 (GPX4) antibody (Cat# BS7323), rabbit-derived anti-Nrf2 antibody (Cat# BS79743), rabbit-derived anti-Glyceraldehyde-3-phosphate dehydrogenase (GAPDH) antibody (Cat# AP0066), and Horseradish peroxidase (HRP)-labeled Goat Anti-Rabbit IgG (Cat# BS20241-Y) were purchased from Bioworld Company (Guangzhou, China). Hematoxylin staining solution (Cat# SI107-02A), Eosin staining solution (Cat# SI107-02B), Benzyldimethylsulfonyl fluoride (Cat# SW106-02), Bicinchoninic Acid Assay Kit (BCA) (Cat# SW201-02), 5× Protein Loading Buffer (Cat# SW116-01), Ultra-sensitive Efficient Chemiluminescence Kit (ECL, Cat# SW134-01), Tween-20 (Cat# SO105-01), DAB Staining Agent (Cat# SI135-01), Bovine Serum Albumin (BSA, Cat# SO110-03), MOPS Electrophoresis Buffer (Cat# SW159-02), Rapid Membrane Transfer Powder (Cat# SW271-02), and Streptozocin Solution (Cat# SC118-01) were purchased from Hunan Saiwen Biotechnology Co., Ltd. (Changsha, China). The DMEM Medium (Cat# C11995500BT) and the RPMI-1640 Medium (Cat# E600028-0500) were purchased from Gibco Company (Shanghai, China). The FBS (Cat# E600051-0500) was purchased from Shanghai Sangong Biotechnology Co., Ltd. (Shanghai, China). The Electron Microscopy Loading Buffer (2.5% glutaraldehyde, Cat# DJ004-15) was purchased from Sichuan Scientist Biotechnology Co., Ltd. (Chengdu, China). The Protein Marker (Cat# 26616) was purchased from Themo Company (Waltham, MA, USA). Neutral Gum (Cat# G8590) and Dimethyl Sulfoxide (DMSO, Cat# 67-68-5) were purchased from Solarbio Company (Beijing, China). The Polyvinylidene Fluoride membrane (PVDF, Cat# PR05509) was purchased from Millipore Company (Billerica, MA, USA). The benzylmethylsulfonyl fluoride (PMSF, Cat# SW106-02) was purchased from Hunan Saiwen Biotechnology Co., Ltd. (Changsha, China). The Radio Immunoprecipitation Assay Lysis Buffer (RIPA, Cat# ES20250518L118) and phosphate buffer solution (PBS, Cat# ES-8006) were purchased from Ecotop Company (Guangzhou, China). The trypsin digestion solution (Cat# ACF107-01) was purchased from Abcell Company (Vancouver, BC, Canada). TRIzol (Cat# 15596026CN) was purchased from Invitrogen Company (Waltham, MA, USA). Sodium Citrate Solution (Cat# C1032) was purchased from Solarbio Company (Beijing, China), 4% Paraformaldehyde (Cat# BL539A) was purchased from Biosharp Company (Beijing, China), Ethanol (Cat# 10009218) was purchased from Sinopharm Chemical Reagent Co., Ltd. (Shanghai, China). The two types of ESCu nanoparticles (TK@ESCu and HA/TK@ESCu) used in this manuscript were provided by Wuhan Zaiyuan Biotechnology Company (Wuhan, China). The Alanine Transaminase (ALT, Cat# C009-1-1) Kit, Glutamic Oxalacetic Transaminase (AST, Cat# C010-1-1) Kit, Total Bilirubin (TBIL, Cat# C019-1-1) Kit, Direct Bilirubin (DBIL, Cat# C019-2-1) Test Kit, Blood Creatinine (Crea, Cat# C011-2-1) Kit and Urea Nitrogen (Urea, Cat# C013-1-1) Kit were purchased from Nanjing Jiancheng Institute of Biotechnology Engineering (Nanjing, China).

### 2.3. Bioinformatics Analysis

The TIMER online database (https://cistrome.shinyapps.io/timer/; accessed on 25 October 2025) was used to query and download the gene transcriptional levels of *FDX1*, *DLAT*, *GPX4,* and *Nrf2* in human liver cancer tissues and corresponding normal liver tissues. This database was also used to analyze the correlations between each pair of the above four genes. Additionally, the GEPIA 2.0 online database (http://gepia2.cancer-pku.cn/#survival; accessed on 25 October 2025) was used to investigate the correlations between the expression of the above four genes and the survival time of liver cancer patients.

### 2.4. CCK-8 Assay for the Proliferation Ability of Liver Cancer Cells

Cells of MHCC97-H and LM3 in the logarithmic growth phase (at which the cell growth rate is exponential) were digested with 0.25% trypsin and collected by centrifugation. The cell concentration was adjusted to 6 × 10^4^ cells/mL with DMEM medium containing 10% FBS, and then 100 μL of the cell suspension was added to each well of a 96-well plate and cultured at 37 °C with 5% CO_2_. After the cells were fully adherent, they were treated with the corresponding drugs. At a specific time after treatment, the drug-containing medium was discarded, and all wells were washed with sterile PBS buffer. Then, 100 μL of fresh DMEM medium containing 10% FBS was added to each well, followed by 10 μL of CCK-8 solution. The reaction was carried out at 37 °C with 5% CO_2_ for 4 h, and the absorbance at 450 nm was detected.

### 2.5. Cell Scratch Assay for Detecting the Migration Ability of Liver Cancer Cells

The MHCC97-H cells and LM3 cells in the logarithmic growth phase (at which the cell growth rate shows an exponential form) were digested and centrifuged, then evenly spread in 6-well plates. When the cell density reached over 95%, a straight line was drawn with a 1 mm scratch bar. The cell debris was washed away with sterile PBS buffer, and the medium was replaced with DMEM high-glucose medium without FBS. The plates were then cultured under 37 °C and 5% CO_2_ conditions. The corresponding drugs were added for specific periods of time (75 or 150 nM of ESCu, named ES1 and ES2; 75 or 150 nM ESCu with 6 μg/mL DDP, named E1D and E2D). Subsequently, the scratch width was photographed under an inverted microscope at 0, 24, and 48 h. The cell migration rate was calculated using the formula: cell migration rate = (scratch width at 0 h − scratch width at 24/48 h)/scratch width at 0 h × 100%.

### 2.6. Measurement of Intracellular Cu^2+^ Concentration

Prepare the test tubes, standard tubes, and blank tubes. Add 150 μL Solution I (Solution I is one of the reagents in the Copper Ion Detection Kit; please refer to Section 2.2) to all the tubes. Count the cells in each treatment group, adjust the concentration of each group to 10^7^ cells/mL, add 1% PMSF to RIPA beforehand, and then incubate for 30 min on ice for lysis. Then centrifuge at 13,000 rpm and 4 °C for 12 min, take the supernatant, and place it on ice for measurement. Add 10 μL of the sample (cell lysate) to the test tubes, 10 μL of the standard to the standard tubes, and 10 μL of ultrapure water to the blank tubes. Let the mixture stand at 37 °C for 5 min. Measure the Optical Density (OD) value in each well and record it as A1. Then add 50 μL Solution II (the Solution II is one of the reagents in the Copper Ion Detection Kit, please refer to Section 2.2) to each tube, let it stand at 37 °C for 5 min, and measure the OD value in each well at 600 nm wavelength and record it as A2. Calculate ΔA = A2 − A1. The calculation formula is: Cu^2+^ (μM) = [(A_test_ − A_blank_)/(A_standard_ − A_blank_)] × standard concentration.

### 2.7. Measurement of Intracellular Fe^2+^ Concentration

Prepare the test tubes, standard tubes, and blank tubes. Add 100 μL of Solution I (the Solution I is one of the reagents in the Ferrous Ion Iron Detection Kit, please refer to Section 2.2) to all the tubes. According to the ratio of bacterial/cell quantity (10^7^): volume of extraction liquid (mL, this is one of the reagents in the Ferrous Ion Detection Kit) of 1:5 (it is recommended to add 2 × 10^7^ cells in 1 mL of extraction liquid (or 10^7^ cells in 0.5 mL of extraction liquid)), perform ice bath ultrasonic disruption of cells (power 200 W, ultrasonic for 5 s, interval 5 s, total time 5 min); then, centrifuge at 13,000 rpm and 4 °C for 12 min, take the supernatant, and place it on ice for measurement. Add 200 μL of the sample to be tested (cell lysate) to the test tubes, 200 μL of the standard sample to the standard tubes, and 200 μL of ultrapure water to the blank tubes. Let the mixture stand at 37 °C for 10 min. For the test tubes, perform vigorous vortexing for 5 min, centrifuge at 12,000 rpm at room temperature for 10 min, and carefully transfer 200 μL of the upper inorganic phase to a 96-well plate. Measure the absorbance at 593 nm and record it as A_test_. Calculate ΔA_test_ = A_test_ − A_blank_. For the standard tubes and blank tubes, directly measure the absorbance at 593 nm and record it as A_standard_ and A_blank_. Calculate ΔA_standard_ = A_standard_ − A_blank_. Based on the concentration (x, μmol/L) of the standard tubes and the absorbance ΔA standard (y, ΔA standard), establish a standard curve. According to the standard curve, substitute ΔA_test_ (y, ΔA_test_) into the formula to calculate the sample concentration (x, μmol/L). The calculation formula is: Fe^2+^ content (μmol/10^7^ cells) = 0.001 x ÷ N, where N is the number of cells.

### 2.8. Measurement of Intracellular ROS

The MHCC97-H cells and LM3 cells in the logarithmic growth phase (where the cell growth rate follows an exponential pattern) were digested with trypsin and centrifuged for collection. They were resuspended in DMEM high-glucose medium containing 10% FBS and evenly spread in 24-well plates. The plates were then placed in a 37 °C, 5% CO_2_ environment for cultivation. After the cells completely adhered to the plate, the corresponding drugs were applied. After the drug treatment was completed, the medium was washed three times without FBS. Subsequently, a mother solution with a concentration of 10 mM DCFH-DA at a volume ratio of 1:1500 was added to the medium without FBS, and the cells were co-incubated for 20 min. The green fluorescence intensity was detected under the irradiation of 488 nm excitation light.

We measured the fluorescence intensity on the microplate reader, setting the excitation wavelength at 488 nm and the emission wavelength at 525 nm. The relative fluorescence units (RFU) values were recorded, and taking the average fluorescence intensity of the negative control group as the reference (set to 1), the fluorescence intensity change factor of each group relative to the negative control group was calculated according to the following formula:

Relative ROS level = (Experimental group RFU − Blank group RFU)/(Negative control group RFU − Blank group RFU).

### 2.9. Measurement of Intracellular LPO

Prepare the test tubes, control tubes, standard tubes, and blank tubes. Add 300 μL Solution I, 200 μL Solution II and 100 μL Solution III (the Solution I, II and III are all components of the reagents in the LPO Detection Kit, please refer to Section 2.2) to each tube. Collect the cells into the centrifuge tube. After centrifugation, discard the supernatant. Add 1 mL of extraction solution to 5 × 10^6^ cells, and use ultrasonic waves to disrupt the cells (in an ice bath, with a power of 200 W, for 3 s each time, with an interval of 7 s, for a total time of 3 min). Centrifuge at 10,000 rpm for 10 min at 4 °C, then take the supernatant and place it on ice for testing.

Then, add 400 μL of the sample to be tested (cell lysate) to the test tube, 400 μL ultrapure water to the control tube, 400 μL of the standard sample to the standard tube, and 400 μL of sample diluent to the blank tube. Mix the solutions and heat them in boiling water for 60 min. Cool them on ice, then centrifuge at 8000 rpm for 10 min. Take 900 μL of the supernatant and transfer it to a 1 mL glass cuvette. Measure the absorbance of each sample at 532 nm and 600 nm, and calculate ΔA = (A532_test_ − A532_control_) − (A600_test_ − A600_control_), ΔA standard = (A532_standard_ − A532_blank_) − (A600_standard_ − A600_blank_). Establish a standard curve based on the concentration (x, nmol/mL) and absorbance ΔA standard (y, ΔA standard) of the standard tubes. Using the standard curve, substitute ΔA (y, ΔA) into the formula to calculate the sample concentration (x, nmol/mL), where the formula is: LPO (nmol/10^4^ cells) = x ÷ cell count (10^4^).

### 2.10. The Morphology of Mitochondria Detected by Transmission Electron Microscopy

Digest the MHCC97-H cells in the logarithmic growth phase (where the cell growth rate follows an exponential pattern) with trypsin, centrifuge at 800 rpm at room temperature for 5 min, resuspend the cells in sterile PBS buffer, and centrifuge again to collect the cells, maintaining the cell count at approximately (0.5–1.0) × 10^6^. Then, fix the cells at 4 °C with 2.5% glutaraldehyde solution for at least 24 h or more, and then add 10% osmium to penetrate the cells. Observe the morphological changes of mitochondria under an 80 keV voltage microscope.

### 2.11. The Protein Expression Levels of Liver Cancer Cells Were Detected by Western Blot

After digesting and centrifuging the liver cancer cells (MHCC97-H or LM3) and the normal liver cell line (LO2) with trypsin, resuspend them in sterile PBS buffer and centrifuge again to collect the cells. Add RIPA cell lysis buffer containing 1% PMSF, and lyse the cells on ice for 30 min. Then centrifuge at 4 °C and 13,000 rpm for 10 min, and take the supernatant and discard the precipitate. Use the micro BCA kit to detect the protein concentration. Adjust the protein mass of the sample to 30 μg with PBS buffer, and adjust the volume to 20 μL. Mix with 5× loading buffer and heat at 100 °C for 6 min. Then perform SDS-PAGE gel electrophoresis under a 100 V voltage. Transfer the protein on the gel to a PVDF membrane at a constant current of 400 mA, and complete the membrane transfer. Place the PVDF membrane in a PBS solution containing 5% skimmed milk at 37 °C for 1 h. Add rabbit anti-FDX1 antibody (GPX4, DLAT, Nrf2, GAPDH antibodies have the same experimental methods as FDX1 antibody) at a specific ratio (parameters provided in the manual) at 4 °C for 12–14 h. Wash 3 times with PBST buffer (PBS buffer with 0.1% Tween-20) for 10 min each time, add HRP-labeled goat anti-rabbit IgG at 37 °C for 1 h, and wash 3 times with PBST buffer for 10 min each time. Finally, use the ECL chemiluminescent solution on the imaging instrument to display the specific protein bands.

### 2.12. Nude Mouse Tumorigenesis and Determination of Tumor Morphological Indicators

Twenty-four 4-week-old nude mice were randomly divided into six groups, labeled as A, B, C, D, E, and F, with four mice in each group. The nude mice lack T cells and can be used to cultivate human tumors and simulate the relevant characteristics of human tumors. All nude mice are housed in SPF-level barrier facilities with a room temperature of 25 °C and 12 h of light per day. They are fed with specially formulated feed. The MHCC97-H cells were cultured, and then 5 × 10^6^ cells were subcutaneously injected into each nude mouse. When the tumor volume was approximately 100 mm^3^, drug intervention was initiated. The nude mice in group A served as the control. The nude mice in group B were intraperitoneally injected with DDP at a concentration of 5 mg/kg every three days. The nude mice in group C were intravenously injected with TK@ESCu at a concentration of 5 mg/kg every two days through the tail vein. The nude mice in group D were intravenously injected with HA/TK@ESCu at a concentration of 5 mg/kg every two days through the tail vein. Group E was a combination of groups B and C, and group F was a combination of groups B and D. During the drug treatment period, the tumor volume and body weight of the nude mice were measured every three days. The experiment was stopped after four drug administrations. For this part of the experiment, we entrusted Hunan SJA Laboratory Animal Co., Ltd. (Changsha, China) to carry it out for us.

### 2.13. Preparation and Hematoxylin-Eosin Staining (HE) of Pathological Sections of Major Organs in Nude Mice

After the cervical dislocation of all nude mice, the fresh organs (heart, liver, spleen, lung, kidney, stomach, intestine, and brain) were removed and placed in 10% neutral formalin fixative solution. They were fixed at room temperature for 24 h. Next, the tissues were rinsed with ultrapure water for 15–30 min, dehydrated with ethanol, and then transparentized with xylene. The tissues were immersed in low-melting-point (56–58 °C) and high-melting-point (60–62 °C) paraffin for thorough wax immersion, and then embedded with high-melting-point paraffin for subsequent sectioning operations.

All the paraffin sections were dewaxed at room temperature for 10 min, repeated twice, then soaked in anhydrous ethanol for 5 min, repeated twice, then immersed in 90% ethanol for 5 min, 75% ethanol for 5 min, and finally rinsed with ultrapure water. Subsequently, they were stained with modified Lillie-Mayer’s hematoxylin for 5 min, rinsed with ultrapure water, washed with 1% acidic differentiation solution for 5 s, rinsed with ultrapure water, and rinsed with ultrapure water again. Finally, the sections were placed in eosin staining solution for 2 min, washed twice with 70% ethanol, and placed under the microscope for direct photography. This part of the experiment was jointly completed by the relevant technicians from Hunan SJA Laboratory Animal Co., Ltd. and us.

### 2.14. Determination of Biochemical Indicators of Liver and Kidney Functions in the Peripheral Blood of Nude Mice

All nude mice were anesthetized by intraperitoneal injection of pentobarbital sodium, and then their eyeballs were used for blood collection.

ALT/AST: Dilute the mouse serum with normal saline 5 times, take 100 μL of the diluted serum and add it to the test tube. Add 500 μL of the matrix solution to both the test tube and the control tube. Incubate at 37 °C in a water bath for 30 min. Then add 500 μL of 2, 4-dinitrophenylhydrazine to both tubes, and add 100 μL of the diluted serum to the control tube. Incubate at 37 °C in a water bath for 20 min. Finally, add 5 mL of 0.4 mol/L sodium hydroxide (NaOH) solution to both tubes and let them stand at room temperature for 5 min. Measure the OD value at a 505 nm wavelength. The absolute OD value = OD value of the test tube − OD value of the control tube. Substitute it into the standard curve to obtain the concentration value (C_0_). The calculation formula is: ALT (U/L) = C_0_ × 0.482 × 5. The determination process of AST is the same as that of ALT.

Urea: First, add 1 mL of Solution I and Solution II to the blank tube, standard tube, and test tube. Then add 20 μL of ultrapure water to the blank tube, 20 μL of urea nitrogen standard solution (10 mM) to the standard tube, and 20 μL of diluted serum to the test tube. Heat in boiling water for 15 min, cool down, and measure the OD value at 520 nm wavelength. The calculation formula is: Urea (mM) = [(A _test_ − A _blank_)/(A _standard_ − A _blank_)] × 10 × 5.

Crea: First, add 180 μL of Solution I to the blank tube, standard tube, and test tube. Then add 6 μL of ultrapure water to the blank tube, 6 μL of the standard sample to the standard tube, and 6 μL of diluted serum to the test tube. Incubate at 37 °C for 5 min, and measure the absorbance value A1 at 546 nm wavelength. Then add 60 μL of Solution II to each tube, incubate at 37 °C for 5 min, and measure the absorbance value A2 at 546 nm wavelength. The calculation formula is: Crea (μM) = [(A _test_ − A _blank_)/(A _standard_ − A _blank_)] × C _standard_ × 5.

This part of the experiment was jointly completed by the relevant technicians from Hunan SJA Laboratory Animal Co., Ltd. and us.

### 2.15. Immunohistochemical Detection of the Expressions of Corresponding Proteins in the Tumor Tissues of Nude Mice

The excised subcutaneous tumors from nude mice were made into paraffin sections. After dewaxing, they were soaked in sodium citrate solution to repair the antigens. After boiling for 30 min, they were cooled at room temperature for 2 h. They were then washed with PBS buffer, and 3% hydrogen peroxide (H_2_O_2_) was added for 10 min for blocking, followed by three washes with PBS for 5 min each. Then, 5% BSA was used for blocking at room temperature for 1 h. The anti-FDX1 antibody (or anti-GPX4 antibody, anti-Nrf2 antibody) was added at a ratio of 1:100 and incubated at 4 °C for 14 h. Then, it was left at room temperature for 30 min, washed three times with PBS for 5 min each, and then the secondary antibody was added for reaction at room temperature for 1 h. The PBS was used for three washes for 5 min each, and then DAB staining solution was added for 5 min at room temperature. The PBS was used for three washes for 5 min each, and then hematoxylin staining was performed for 20 s, followed by rinsing with ultrapure water for 5 min. After gradient ethanol dehydration, neutral gum was added for covering, and it was left at room temperature for 2 h before taking pictures.

This part of the experiment was jointly completed by the relevant technicians from Hunan SJA Laboratory Animal Co., Ltd. and us.

### 2.16. Statistical Analysis

All quantitative experiments were repeated 3 times or more. The measurement data were expressed as the mean ± standard deviation (Mean ± SD). The data were statistically analyzed by Graphpad prism 8.0 software. The differences between the two groups of measurement data were analyzed using Student’s *t*-test. A difference was considered statistically significant if *p* < 0.05. Statistical significance was indicated as follows: * *p* < 0.05, ** *p* < 0.01, *** *p* < 0.001, **** *p* < 0.0001. The term “ns” denotes no statistical significance.

## 3. Results

### 3.1. The Bioinformatics Analysis of the Expression and the Relationship with Prognosis of the Cross-Marker Between Ferroptosis and Cuproptosis in HCC

**Figure 1:**
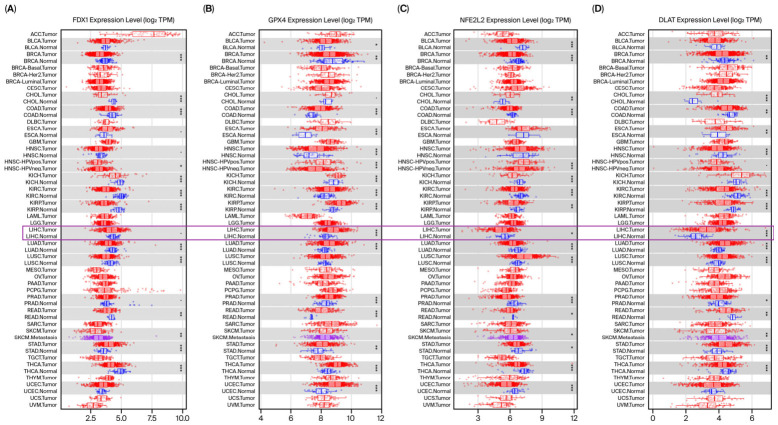

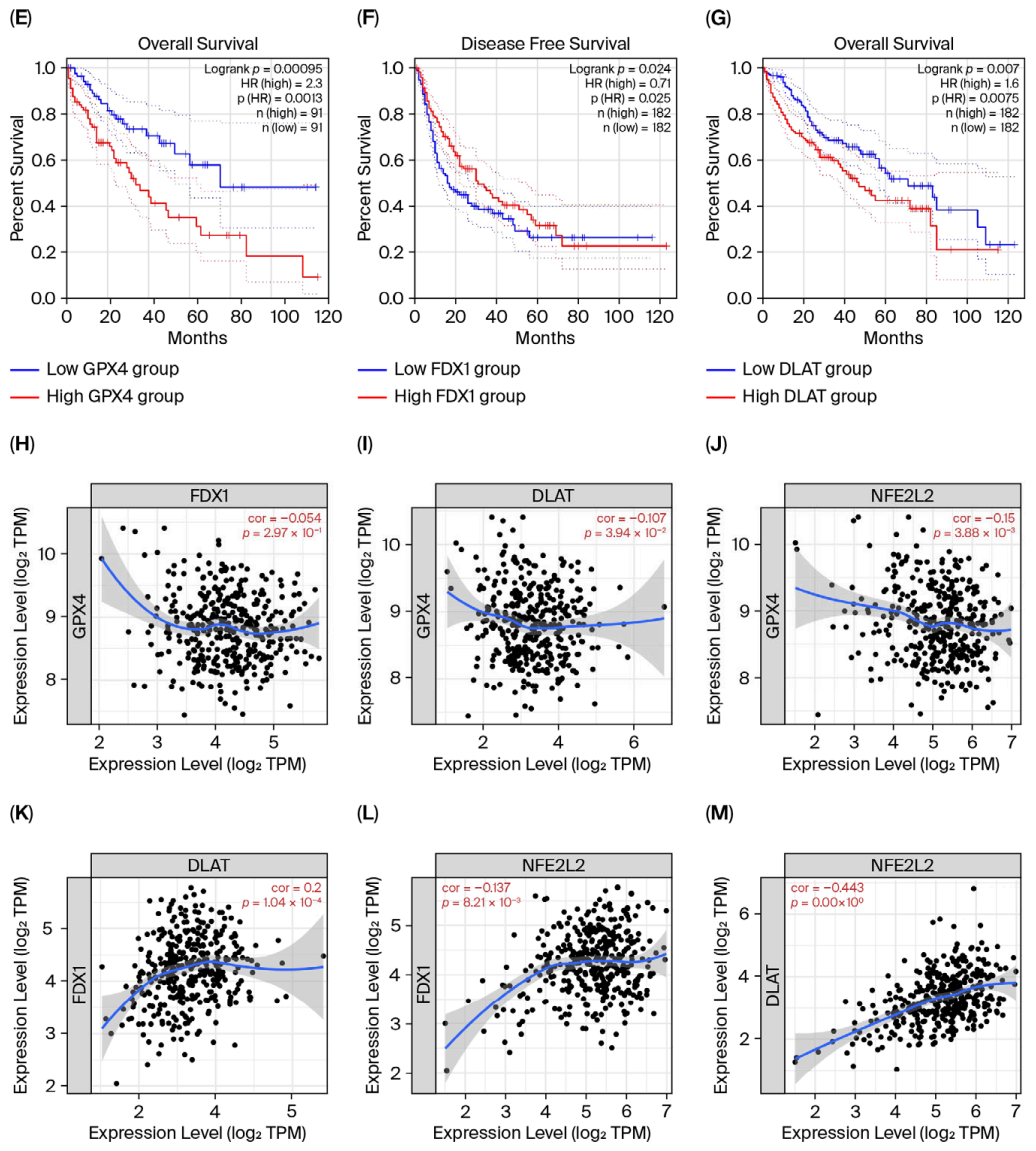
Bioinformatics analysis of the expression of cross-related markers of ferroptosis and cuproptosis in HCC patients and their correlation with patient survival time. (**A**–**D**) The expression of FDX1, GPX4, Nrf2, and DLAT in HCC tumor tissues and normal liver tissues; data from the TIMER online database. (**E**–**G**) The correlation between the expression levels of GPX4, FDX1, and DLAT and the survival time of HCC patients; data from the GEPIA 2.0 database. (**H**–**M**) The correlation analysis between each pair of the four markers (FDX1, GPX4, Nrf2, and DLAT); data from the TIMER online database. * *p* < 0.05, ** *p* < 0.01, *** *p* < 0.001.

The occurrence of cuproptosis and ferroptosis both requires the activation of intracellular reactive oxygen species to induce oxidative stress. The molecules closely related to this process mainly include FDX1, GPX4, Nrf2, and DLAT. The TIMER software and GEPIA 2.0 software were used to analyze the expression of these four indicators in HCC patients and their correlations with survival time. We found that compared with the control group, GPX4 and DLAT were significantly elevated in HCC patients, while Nrf2 was overall expressed at lower levels in all HCC patients than in the control group, but there were also more patients with high expression. There was no statistical difference in the expression of FDX1 between HCC patients and the control group (Figure 1A–D, * *p* < 0.05, *** *p* < 0.001). The overall survival time of HCC patients with high expression of GPX4 was significantly lower than that of those with low expression (Figure 1E, *p* = 0.0013), the disease-free survival time of HCC patients with high expression of FDX1 was significantly higher than that of those with low expression (Figure 1F, *p* = 0.025), and the total survival time of HCC patients with high expression of DLAT was significantly lower than that of those with low expression (Figure 1G, *p* = 0.0075). There was a correlation between these indicators, suggesting the cross-correlation between the cuproptosis and ferroptosis signaling pathways in hepatocellular carcinoma cells (Figure 1H–M).

### 3.2. ESCu Can Induce the Death of Liver Cancer Cells In Vitro and Enhances the Anti-Tumor Effect of Cisplatin

**Figure 2:**
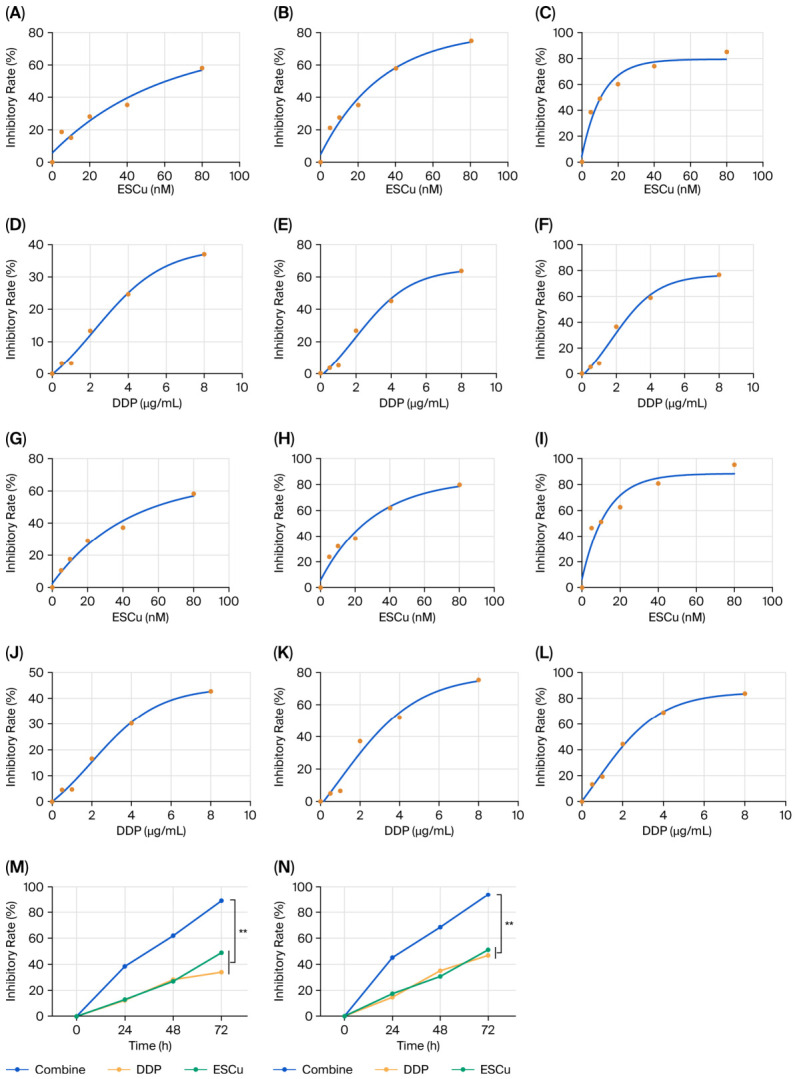

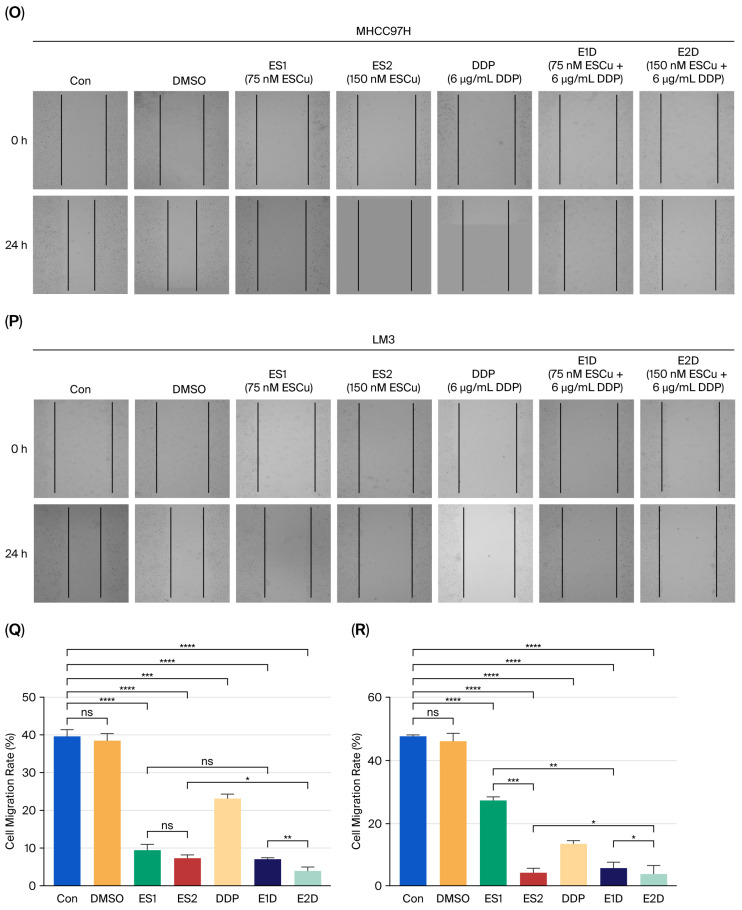
The in vitro synergistic anti-tumor effect of ESCu and DDP. (**A**–**C**) Inhibition rates of MHCC97-H cells treated with different concentrations of ESCu (0/5/10/20/40/80 nM) after 24/48/72 h. (**D**–**F**) Inhibition rates of MHCC97-H cells treated with different concentrations of DDP (0/0.5/1/2/4/8 μg/mL) after 24/48/72 h. (**G**–**I**) Inhibition rates of LM3 cells treated with different concentrations of ESCu (0/5/10/20/40/80 nM) after 24/48/72 h. (**J**–**L**) Inhibition rates of LM3 cells treated with different concentrations of DDP (0/0.5/1/2/4/8 μg/mL) after 24/48/72 h. (**M**) Inhibition rates of MHCC97-H cells treated with 10 nM ESCu and 2 μg/mL DDP alone or in combination after 24/48/72 h. (**N**) Inhibition rates of LM3 cells treated with 10 nM ESCu and 2 μg/mL DDP alone or in combination after 24/48/72 h. (**O**–**Q**) Migration experiment graphs of MHCC97-H cells treated with different conditions. (**P**,**R**) Migration experiment graphs of LM3 cells treated with different conditions. The measurement data in this figure are all from 3 independent repeated tests, expressed as the mean ± standard deviation (X ± SD), and the statistical differences were determined by an independent sample *t*-test. ES1: 75 nM ESCu, ES2: 150 nM ESCu, E1D: 75 nM ESCu + 6 μg/mL DDP, E2D: 150 nM ESCu + 6 μg/mL DDP. * *p* < 0.05, ** *p* < 0.01, *** *p* < 0.001, **** *p* < 0.0001, ns indicates no statistical significance.

To investigate the inhibitory effects of ESCu and DDP on liver cancer cells in vitro and their synergistic effects, the CCK-8 and cell scratch assays were used to evaluate the inhibitory effects of the two on liver cancer cells. The MHCC97-H or LM3 cells were treated with 0/5/10/20/40/80 nM or 0/0.5/1/2/4/8 μg/mL of DDP, and the inhibition rates of ESCu and DDP on the two types of liver cancer cells at 24 h, 48 h, and 72 h were measured, expressed as the half-maximal inhibitory concentration (IC50). We found that the inhibitory effects of ESCu and DDP on liver cancer cells were both dose-dependent and time-dependent. The IC50 of ESCu for MHCC97-H and LM3 cells decreased with time (Figure 2A–C,G–I, Table 1), and the IC50 of DDP for 97H and LM3 cells at 48 h and 72 h were similar (Figure 2D–F,J–L, Table 2). The DDP treatment of 97H and LM3 cells for 24 h was not sufficient to effectively inhibit the growth of liver cells (IC50 > 8 μg/mL, Figure 2D,J). To investigate the inhibitory effect of the combined use of the two on liver cancer cells, we treated the two types of liver cancer cells with 10 nM ESCu and 2 μg/mL DDP. At these concentrations, both ESCu and DDP had relatively low inhibitory rates on liver cancer cells when used alone (the inhibition rate was less than 50% after 72 h of treatment), but the combined use significantly enhanced the inhibitory effect on liver cancer cells (*p* < 0.01, Figure 2M,N), confirming that ESCu and DDP can effectively kill liver cancer cells and exert a synergistic effect. Subsequently, to study the effects of ESCu and DDP on the migration ability of liver cancer cells, 75 nM and 150 nM concentrations of ESCu (named ES1 and ES2, respectively) and 6 μg/mL of DDP were used for scratch assays. The results showed that ESCu could significantly inhibit the migration of liver cancer cells, and its inhibitory effect was stronger than that of DDP; while when ESCu and DDP were used in combination, the inhibitory effect on the migration of liver cancer cells was better (Figure 2O–R). These data indicate that ESCu and DDP have a synergistic effect and can enhance the in vitro anti-tumor effect by interacting with each other.

### 3.3. ESCu and Cisplatin Can Synergistically Induce Oxidative Stress in Liver Cancer Cells

**Figure 3:**
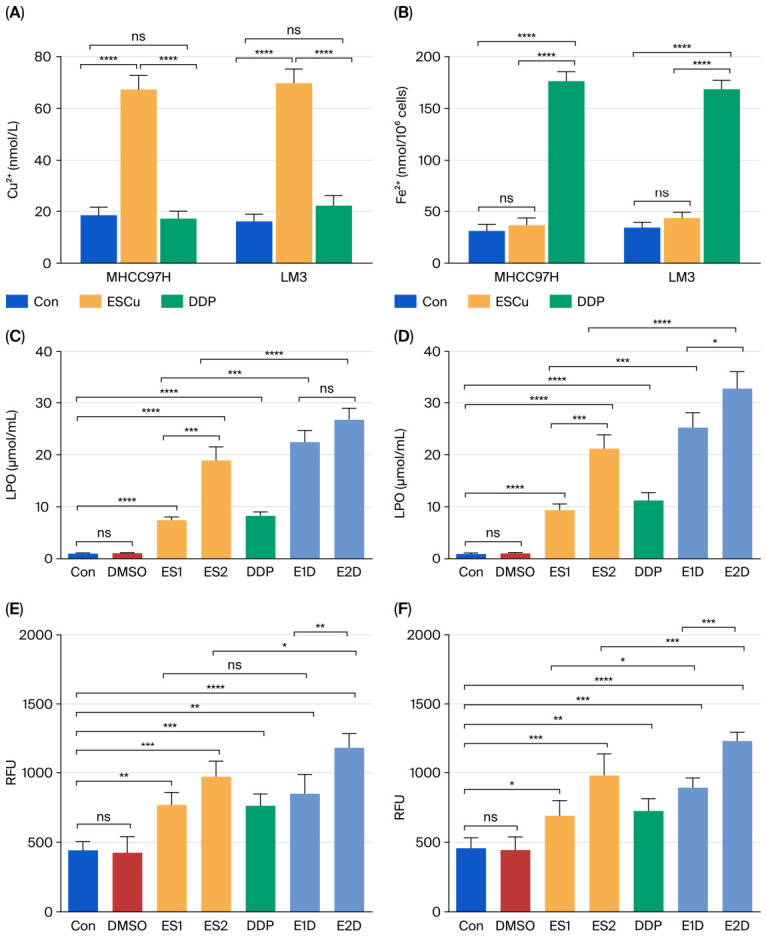
ESCu induces cuproptosis and enhances the effect of ferroptosis caused by DDP in liver cancer cells. (**A**) The intracellular Cu^2+^ concentration in MHCC97-H and LM3 cells under different treatment conditions was detected using a copper ion detection kit. (**B**) The intracellular Fe^2+^ concentration in MHCC97-H and LM3 cells under different treatment conditions was detected using a ferrous ion detection kit. (**C**,**D**) The intracellular LPO levels in MHCC97-H and LM3 cells under different treatment conditions were detected using an LPO detection kit. (**E**,**F**) The relative fluorescence units (RFU) values from liver cancer cells in different treatment conditions were detected using a microplate system. * *p* < 0.05, ** *p* < 0.01, *** *p* < 0.001, **** *p* < 0.0001, ns indicates no statistical significance.

Elesclomol acts as a carrier for Cu^2+^, enabling the transport of Cu^2+^ into cells and inducing cuproptosis. To verify whether ESCu can induce cuproptosis in liver cancer cells and whether there is a synergistic effect between ESCu and DDP, the concentrations of Fe^2+^ and Cu^2+^ within liver cancer cells were examined. Compared with the control group, ESCu significantly increased the concentration of Cu^2+^ in cells in vitro, but did not increase the concentration of Fe^2+^; DDP significantly increased the content of Fe^2+^ in cells, but did not increase the content of Cu^2+^; the combined application of ESCu and DDP significantly increased the concentrations of Fe^2+^ and Cu^2+^ within cells (Figure 3A,B). Subsequently, we also examined the ability of Fe^2+^ and Cu^2+^ to cause oxidative stress and found that both ESCu and DDP could promote the levels of ROS and LPO within liver cancer cells (Figure 3C–F).

The RFU values from liver cancer cells in different treatment conditions showed that both ESCu and DDP could significantly increase the ROS levels within liver cancer cells. When used together, they can synergistically stimulate even higher levels of ROS (Figure 3E,F). These data indicate that both ESCu and DDP can significantly induce oxidative stress in liver cancer cells, suggesting that they may have a synergistic effect.

### 3.4. ESCu and Cisplatinum Respectively Induce Cuproptosis and Ferroptosis, Thereby Jointly Promoting Oxidative Stress in Liver Cancer Cells

**Figure 4:**
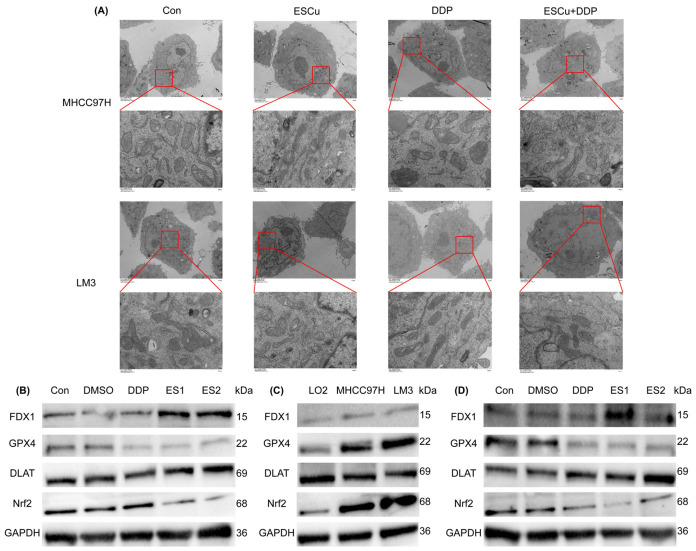
ESCu and DDP induce cuproptosis and ferroptosis to generate oxidative stress. (**A**) The ultrastructural changes of mitochondria in MHCC97-H and LM3 cells under different treatment conditions were detected by transmission electron microscopy. (**B**–**D**) The protein expression of FDX1, GPX4, DLAT, and Nrf2 in MHCC97-H and LM3 cells under different treatment conditions was detected by Western blot. H. The protein expression of FDX1, GPX4, DLAT, and Nrf2 in LO2 cells, MHCC97-H, and LM3 cells was detected by Western blot.

To deeply explore the molecular mechanism underlying the synergistic effect of ESCu and cisplatin in vitro, the mitochondrial morphological characteristics and the expression levels of related proteins in ferroptosis and cuproptosis were detected. Our results showed that ESCu alone or in combination with DDP could induce liver cancer cells to exhibit shrunken mitochondria (Figure 4A). From the perspective of molecular mechanisms, our data revealed that ESCu could upregulate the expression of FDX1 in liver cancer cells and downregulate the expression of GPX4 and Nrf2 (Figure 4B–D). These results indicate that ESCu can regulate the expression of molecules related to signaling pathways that are cross-linked with ferroptosis and cuproptosis in liver cancer cells.

### 3.5. ESCu Nanoparticles Have Good Biological Safety

**Figure 5:**
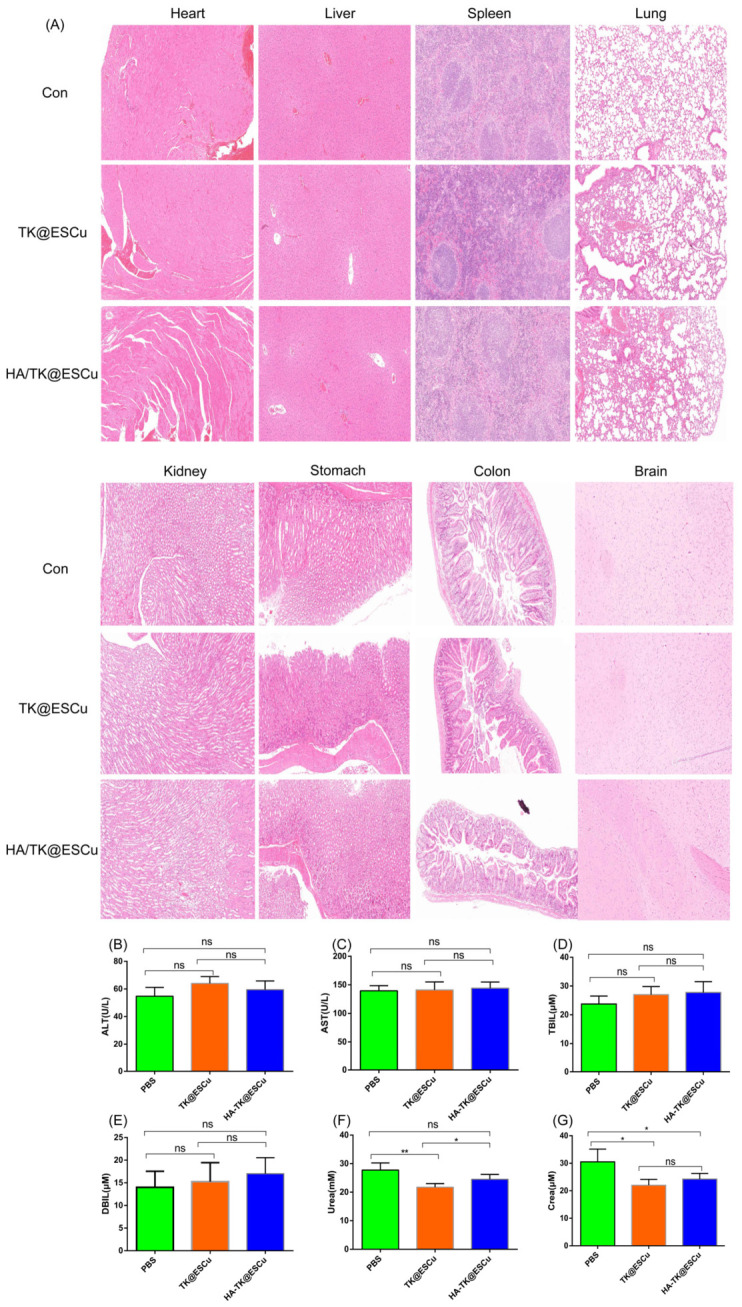
Biological safety detection of ESCu nanoparticles. (**A**) The pathological damage changes in the main organs of nude mice were detected by using Hematoxylin-Eosin (HE) staining. (**B**–**G**) Analysis of serum biochemical indicators of each treatment group (4 mice per group): (**B**) Alanine aminotransferase (ALT); (**C**) Aspartate aminotransferase (AST); (**D**) Total bilirubin (TBIL); (**E**) Direct bilirubin (DBIL); (**F**) Urea; (**G**) Creatinine (Crea). The quantitative data (**B**–**G**) were all obtained from 4 independent repeated tests, expressed as the mean ± standard deviation (X ± SD), and statistical differences were determined by an independent sample *t*-test. * *p* < 0.05, ** *p* < 0.01, ns indicates no statistical significance.

To detect the potential organ toxicity of TK@ESCu and HA/TK@ESCu in vivo, we conducted sectioning and HE staining tests on the hearts, livers, spleens, lungs, stomachs, intestines, brains, and other organs of each group of experimental mice. We found that compared with the PBS group, neither TK@ESCu nor HA/TK@ESCu caused significant pathological changes in the organs of the experimental mice. No inflammatory phenomena such as bleeding, necrosis, or cell fragmentation were observed under the microscope (Figure 5A). Subsequently, we also measured the levels of creatine kinase, alanine aminotransferase, aspartate aminotransferase, creatinine, and urea in the peripheral blood of each group of experimental mice. We found that TK@ESCu and HA/TK@ESCu only caused a slight decrease in creatinine and urea levels in the experimental mice (Figure 5B–E). These data indicate that the ESCu nanoparticles we prepared have good biocompatibility and very low cytotoxicity to normal tissues.

### 3.6. The High Feedback Expression of Nrf2 May Be the Key Factor Restricting the Synergistic Anti-Tumor Effect of ESCu in Combination with Cisplatin In Vivo

**Figure 6:**
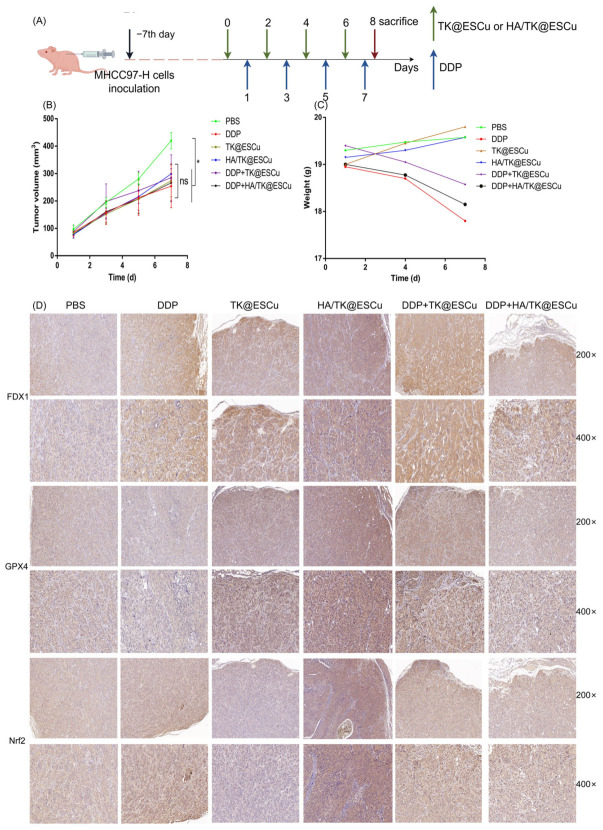
In vivo anti-hepatocellular carcinoma effect of ESCu nanoparticles combined with DDP**.** (**A**) The procedure for animal experiments, divided into groups as shown in the figure; 4 nude mice were randomly assigned to each group. Each nude mouse was injected with 5 × 10^6^ MHCC97-H cells. Approximately 7 days later, the tumor volume reached 100 mm^3^. This was recorded as day 0. Then, the corresponding group of nude mice was intravenously injected with 5 mg/kg TK@ESCu or HA/TK@ESCu at days 0/2/4/6, and intraperitoneally injected with 5 mg/kg DDP at days 1/3/5/7. The control group was intravenously injected with an equal volume of sterile PBS at days 0/2/4/6, and intraperitoneally injected with an equal volume of sterile PBS at days 1/3/5/7. On the 8th day, the nude mice were decerebrated by cervical dislocation, and the tumor tissue was removed to obtain major organs (for biological safety testing). (**B**) Tumor volume measurement, the volume calculation formula is V = ab^2^/2 mm^3^, where a and b are the long diameter and short diameter of the tumor, and the tumor volume of each nude mouse was measured 3 times and averaged. (**C**) Measurement of mouse body weight: the body weight of each nude mouse was measured 3 times and averaged. (**D**) Tumor tissue sections of nude mice were subjected to immunohistochemical staining to detect the expression levels of FDX1, GPX4, and Nrf2 in the tumor tissue, and the groups were the same as before. The measurement data (**B**,**C**) were from 3 independent repeated tests, expressed as the mean ± standard deviation (X ± SD), and the statistical differences were determined by an independent sample *t*-test. * *p* < 0.05, ns indicates no statistical significance.

To study the in vivo synergistic anti-tumor effects of TK@ESCu and HA/TK@ESCu with DDP, after MHCC97-H cells formed tumors subcutaneously in nude mice, different groups of experimental mice were injected with TK@ESCu or HA/TK@ESCu at a dose of 5 mg/kg. The control group was injected with the same volume of PBS. The final concentration of DDP was 5 mg/mL, and each injection was 100 μL. All drugs were injected every 2 days, and the body weight and tumor volume of nude mice were measured every 3 days. The results showed that both TK@ESCu and HA/TK@ESCu could effectively inhibit the growth of subcutaneous tumors, and the inhibitory effect was not statistically different from that of DDP. However, the tumor suppression effect of the group combining TK@ESCu or HA/TK@ESCu with DDP was not statistically different from that of using nanoparticles or DDP alone (Figure 6A–C). Immunohistochemical analysis of the tumor tissues of each group of nude mice revealed that TK@ESCu and HA/TK@ESCu could induce the expression of FDX1 in tumor tissues, and DDP could inhibit the expression level of GPX4 in nude mouse tumor tissues. However, the expression of Nrf2 in tumor tissues that were treated with TK@ESCu or HA/TK@ESCu with DDP had a certain degree of feedback upregulation (Figure 6D). These data suggested that the high expression of Nrf2 may be an important reason for the lack of synergy between ESCu and DDP in vivo. High levels of reducing substances can effectively neutralize high concentrations of ROS, and thereby counteract the ROS produced synergistically by ferroptosis and cuproptosis.

## 4. Discussion

Chemotherapy and targeted therapies inevitably cause additional liver damage, resulting in numerous toxic side effects. Because HCC is difficult to diagnose early, drug therapy is ineffective, and patients’ liver function has already been severely compromised. This presents serious challenges to improving therapy results and prolonging HCC patients’ lives. In animal models, the two nanomedicines TK@ESCu and HA/TK@ESCu showed favorable biosafety profiles. There were no additional notable harmful side effects, with the exception of causing pulmonary inflammation and slight changes in renal function markers. Notably, their therapeutic efficacy did not differ significantly from DDP, and they had a minor impact on the liver without compromising hepatic function. The primary mechanism via which ESCu nanoparticles suppress tumor cells is copper-induced cell death. Given the liver’s high copper concentration, using copper-induced cell death to treat liver cancer is an extremely promising new treatment technique.

DDP, a routinely used chemotherapeutic drug for liver cancer, is particularly important in patients who have failed to respond to targeted therapy and immunotherapy. One of DDP’s primary strategies for killing tumor cells is ferroptosis, which exerts its antitumor impact by producing lipid peroxides on the cell membrane. However, people with liver cancer rapidly develop resistance to DDP. The unusually high expression of products related to the tumor cell’s reduction system has a significant impact on LPO formation. The aberrant activation of the Nrf2 signaling pathway is the primary mechanism that limits cellular oxidative damage. Upon activation, Nrf2 increases the creation of large amounts of GSH within cells, which undergoes redox interactions with ROS products, providing antioxidant protection to normal tissue cells [26]. For instance, Yang et al. reported that Maresin1 significantly alleviates lipopolysaccharide (LPS)-induced acute liver injury, primarily by activating the Nrf2/GPX4 signaling pathway within hepatocytes and thereby inhibiting LPO production [27]. However, this mechanism confers drug resistance in tumor cells, as demonstrated in a study on aristolochic acids (AAs)-induced hepatocellular carcinoma. AAs promote tumor growth by inhibiting ferroptosis in hepatocellular carcinoma cells through regulation of the p53/GADD45A/NRF2/SLC7A11 signaling axis [28]. In this investigation, we observed that DDP-treated MHCC97-H and LM3 cells had lower protein expression levels of both GPX4 and FTH1, although a feedback-induced increase in Nrf2 expression counterbalanced this. This suggests the formation of substantial numbers of decreased products within hepatocellular cancer cells, which will inevitably result in DDP tolerance.

The signaling pathways of copper-induced cell death and ferroptosis exhibit partial overlap, both sharing the common mechanism of inducing intracellular oxidative stress to kill cells. Consequently, the expression levels of intracellular reduction systems inevitably influence the efficacy of copper-induced cell death. High Nrf2 expression causes cells to create significant levels of GSH, which counteract the oxidative damage caused by ESCu [29]. Under ESCu stress selection, tumor cells gradually develop resistance to ESCu via the Nrf2/GSH signaling pathway. In this study, whether hepatocellular carcinoma cells were treated in vitro with ESCu or ESCu nanoparticles were administered in vivo to cancer-bearing mice, FDX1 expression levels were raised in tumor cells, while Nrf2 expression was simultaneously upregulated. This conclusion is consistent with the effects observed with DDP. Crucially, ESCu failed to induce oxidative stress even under high GSH conditions despite generating substantial ROS, suggesting this mechanism may represent a key pathway underpinning ESCu resistance in hepatocellular carcinoma cells. Reports in this area support our findings by showing that ESCu uses feedback mechanisms to adversely regulate Nrf2 protein stability while carrying out its biological responsibilities. This leads to the upregulation of expression in both the glutathione S-transferase modulating subunit (GCLM) and the glutathione S-transferase catalytic subunit (GCLC). GCLM and GCLC are rate-limiting enzymes in GSH synthesis. The solute carrier family 25 member 39 (*SLC25A39*) transporter carries highly expressed GSH into mitochondria. GSH entering the mitochondria counteracts copper-induced cell death by neutralizing ESCu-induced reactive oxygen species, while Cu^2+^ undergoes redox reactions within the mitochondria [30]. GSH plays a critical role in modulating cellular resistance to copper-induced cell death, as Liu et al. showed in a mouse pancreatic cancer model that genetically inhibiting the Nrf2-GSH signaling pathway dramatically increased the efficacy of antitumor therapy [31]. Our conclusions are also supported by reports from non-tumor domains. As an example, by binding to the ubiquitin ligase Keap1 and controlling the Nrf2 signaling pathway, circSpna2, a member of the non-coding RNA family, can prevent copper-induced death in brain neurons after trauma. Similar to this mechanism, increased expression of Nrf2 triggers the synthesis of GSH, which uses reductive mechanisms to protect cells from oxidative stress [32].

Although the inhibitory effect of ESCu nanoparticles combined with DDP on liver cancer in vivo showed no statistically significant difference compared to ESCu or DDP alone, this study revealed that ESCu nanoparticles exhibit high biosafety in vivo. There was no discernible tissue or organ toxicity, with the exception of causing moderate pulmonary inflammation and minor changes in renal function. This discovery highlights ESCu’s significant clinical translational potential for upcoming antitumor treatments. Building on this foundation, the concurrent administration of Nrf2 inhibitors and ESCu therapy for liver cancer appears to be an optimal treatment strategy. This technique may effectively prevent drug resistance caused by Nrf2 activation, according to current scientific research. Zhang et al. reported that a curcumin derivative effectively inhibits the proliferation of cervical cancer cells. Mechanistic studies demonstrated that this derivative strongly inhibited the intracellular Nrf2 signaling pathway while increasing FDX1 expression. This not only reduced glutathione (GSH) production but also prevented nicotinamide adenine dinucleotide phosphate (NADPH) action, lowering intracellular NADPH levels, further decreasing intracellular reducing agents, which inhibits cervical cancer cell invasion and migration [33]. Furthermore, Qiao et al. designed nanoparticles incorporating Cu^2+^ and quinone compounds, wherein Cu^2+^ induces oxidative stress within cells, while the quinone ligands undergo Michael addition reactions with intracellular glutathione (GSH) and catalase-like reactions, depleting GSH and suppressing Nrf2 protein expression levels. Furthermore, the severe oxidative responses change the tumor microenvironment, allowing immune cells to infiltrate tumor tissues and increasing the body’s antitumor immune response efficacy [34]. Our research further revealed that ESCu treatment induced alterations in multiple immune-related genes within MHCC97-H cells, including changes in the transcriptional levels of certain chemokines and their receptors. Additionally, ESCu promoted the upregulation of human leukocyte antigen-I (HLA-I) class gene transcription in MHCC97-H cells, suggesting that ESCu possesses the potential to remodel the immune microenvironment. Future research may examine the use of ESCu in conjunction with immunotherapy for different types of solid tumors, but further research is needed to determine the exact pathways. Additionally, there was no statistically significant difference between the two types of ESCu nanoparticles synthesized in this experiment in terms of their in vivo inhibitory effects on liver cancer. This implies that ROS-responsive materials are already adequate to allow ESCu nanoparticles to build up in the in vivo tumor microenvironment. According to Guo et al., when nanoparticles encapsulated ESCu, they effectively reduced bladder cancer development in vivo despite having only ROS responsiveness. Similarly, this suggests that adding more targeting molecules to the surface of the nanoparticle might not be required [15].

It must be pointed out that this study is the first to explore the therapeutic effect of ESCu nanomedicine in liver cancer. Although ESCu nanomedicine can effectively inhibit the proliferation of liver cancer cells in vitro and in vivo, the combined use of ESCu nanomedicine and DDP did not show better efficacy than the single one. This is a limitation of this study and a challenge that needs to be overcome in future research. The abnormal activation of the Nrf2 signaling pathway under high-intensity oxidative stress stimulation is likely to be an important cause of this phenomenon, but the specific molecular mechanism still needs further investigation. In addition, what factors regulate the abnormal activation of the Nrf2 signal pathway, whether other redox-related signaling pathways are also activated, and whether the multidrug resistance genes that cause primary resistance of tumor cells to multiple drugs are also involved in this process are all questions that require more in-depth research.

## 5. Conclusions

In conclusion, our work shows that copper-induced death technology is therapeutically effective in nanomedicine, particularly for hepatocellular cancer. ESCu significantly inhibits the proliferation of hepatocellular carcinoma cells both in vivo and in vitro, and exhibits favorable biological safety. This study shows great potential for use in different cancer therapies and presents a fresh strategy for creating highly effective, low-toxicity medicines against liver cancer. It creates a novel model for improving liver cancer therapy outcomes by combining nanotechnology with copper-induced cell death.

## Figures and Tables

**Table 1:** The IC50 values of ESCu in MHCC97-H and LM3 cells after different time treatments.

	24 h	48 h	72 h
MHCC97-H	61.3	30.4	10.1
LM3	56.2	25.7	10.0

**Table 2:** The IC50 values of DDP in MHCC97-H and LM3 cells after different time treatments (μg/mL).

	24 h	48 h	72 h
97H	>8	4.50	3.16
LM3	>8	3.53	2.67

## Data Availability

The original contributions presented in this study are included in the article. Further inquiries can be directed to the corresponding author.

## Abbreviations

Abbreviations	Full English names
ALT	alanine transaminase
AST (other name: GOT)	glutamic oxalacetic transaminase
BCA	bicinchoninic acid assay kit
BSA	bovine serum albumin
CCK-8	cell counting kit-8
Crea	creatinine
DBIL	direct bilirubin
DCFH-DA	2′,7′-dichlorodihydrofluorescein diacetate
DDP	cisplatin
DLAT	dihydrolipoamide S-acetyltransferase
DMEM	dulbecco’s modified eagle medium
DMSO	dimethyl sulfoxide
ECL	efficient chemiluminescence kit
ES	elesclomol
ESCu	esclomol-copper chloride
FBS	fetal bovine serum
FDX1	ferredoxin 1
GAPDH	glyceraldehyde-3-phosphate dehydrogenase
GCLC	glutathione S-transferase catalytic subunit
GCLM	glutathione S-transferase modulating subunit
GPX4	glutathione peroxidase 4
GSH	glutathione
HA	hyaluronic acid
HCC	hepatocellular carcinoma
HE	hematoxylin-eosin staining
HLA-I	human leukocyte antigen-I
HRP	horseradish peroxidase
IC50	half-maximal inhibitory concentration
LPO	lipid peroxide
LPS	lipopolysaccharide
Nrf2	nuclear factor erythroid 2-related factor 2
OD	optical density
PBS	phosphate buffer solution
PMSF	benzylmethylsulfonyl fluoride
PD-L1	programmed death-ligand 1
PVDF	polyvinylidene fluoride membrane
RFU	relative fluorescence units
RIPA	radio immunoprecipitation assay lysis buffer
ROS	reactive oxygen species
RPMI-1640	roswell park memorial institute 1640 medium
TBIL	total bilirubin
TK	thioketal
Urea	urea nitrogen

## References

[1] Yan S., Chen L., Zhuang H., Yang H., Yang Y., Zhang N., Liu R. (2024). HDAC Inhibition Sensitize Hepatocellular Carcinoma to Lenvatinib via Suppressing AKT Activation. Int. J. Biol. Sci..

[2] Wang K., Xiang Y., Yu H., Cheng Y., Liu Z., Qin Y., Shi J., Guo W., Lu C., Zheng Y. (2024). Adjuvant Sintilimab in Resected High-Risk Hepatocellular Carcinoma: A Randomized, Controlled, Phase 2 Trial. Nat. Med..

[3] Jin Z., Zhong B., Chen J., Zhu H., Sun J., Yin G., Ge N., Luo B., Ding W., Li W. (2023). Real-World Efficacy and Safety of TACE plus Camrelizumab and Apatinib in Patients with HCC (CHANCE2211): A Propensity Score Matching Study. Eur. Radiol..

[4] Lu Y., Zhu J., Zhang Y., Li W., Xiong Y., Fan Y., Wu Y., Zhao J., Shang C., Liang H. (2024). Lactylation-Driven IGF2BP3-Mediated Serine Metabolism Reprogramming and RNA m6A-Modification Promotes Lenvatinib Resistance in HCC. Adv. Sci..

[5] Tan J., Fan W., Liu T., Zhu B., Liu Y., Wang S., Wu J., Liu J., Zou F., Wei J. (2023). TREM2+ Macrophages Suppress CD8+ T-Cell Infiltration after Transarterial Chemoembolisation in Hepatocellular Carcinoma. J. Hepatol..

[6] Sun T., Sun B., Cao Y., Liu J., Chen J., Liang B., Zheng C., Kan X. (2023). Synergistic Effect of OK-432 in Combination with an Anti-PD-1 Antibody for Residual Tumors after Radiofrequency Ablation of Hepatocellular Carcinoma. Biomed. Pharmacother. Biomed. Pharmacother..

[7] Tsvetkov P., Coy S., Petrova B., Dreishpoon M., Verma A., Abdusamad M., Rossen J., Joesch-Cohen L., Humeidi R., Spangler R. (2022). Copper Induces Cell Death by Targeting Lipoylated TCA Cycle Proteins. Science.

[8] Hasinoff B., Yadav A., Patel D., Wu X. (2014). The cytotoxicity of the anticancer drug elesclomol is due to oxidative stress indirectly mediated through its complex with Cu(II). J. Inorg. Biochem..

[9] Liu X., Qu H., Li J., Sun X., Wang Z., Wang D., Bai X., Li X. (2025). p53 enhances elesclomol-Cu-induced cuproptosis in hepatocellular carcinoma via FDXR-mediated FDX1 upregulation. Front. Oncol..

[10] Del Bello F., Pellei M., Bagnarelli L., Santini C., Giorgioni G., Piergentili A., Quaglia W., Battocchio C., Iucci G., Schiesaro I. (2022). Cu(I) and Cu(II) Complexes Based on Lonidamine-Conjugated Ligands Designed to Promote Synergistic Antitumor Effects. Inorg. Chem..

[11] Modica-Napolitano J., Murray M., Thibault J., Haley-Read J., Nixdorf L., Shanahan B., Iacovella N., Reyes C. (2024). The In Vitro Cytotoxic Effect of Elesclomol on Breast Adenocarcinoma Cells Is Enhanced by Concurrent Treatment with Glycolytic Inhibitors. Cancers.

[12] Li Y., Yang J., Zhang Q., Xu S., Sun W., Ge S., Xu X., Jager M., Jia R., Zhang J. (2022). Copper Ionophore Elesclomol Selectively Targets GNAQ/11-Mutant Uveal Melanoma. Oncogene.

[13] Gao W., Huang Z., Duan J., Nice E., Lin J., Huang C. (2021). Elesclomol Induces Copper-Dependent Ferroptosis in Colorectal Cancer Cells via Degradation of ATP7A. Mol. Oncol..

[14] O’Day S., Eggermont A., Chiarion-Sileni V., Kefford R., Grob J., Mortier L., Robert C., Schachter J., Testori A., Mackiewicz J. (2013). Final Results of Phase III SYMMETRY Study: Randomized, Double-Blind Trial of Elesclomol plus Paclitaxel versus Paclitaxel Alone as Treatment for Chemotherapy-Naive Patients with Advanced Melanoma. J. Clin. Oncol. Off. J. Am. Soc. Clin. Oncol..

[15] Guo B., Yang F., Zhang L., Zhao Q., Wang W., Yin L., Chen D., Wang M., Han S., Xiao H. (2023). Cuproptosis Induced by ROS Responsive Nanoparticles with Elesclomol and Copper Combined with αPD-L1 for Enhanced Cancer Immunotherapy. Adv. Mater..

[16] Chen C., Zhao S., Karnad A., Freeman J. (2018). The Biology and Role of CD44 in Cancer Progression: Therapeutic Implications. J. Hematol. Oncol..

[17] Ke B., Wu X., Yang Q., Huang Y., Wang F., Gong Y., Liu J., Shi L. (2019). Yi-Qi-Yang-Yin-Tian-Sui-Fang Enhances Cisplatin-Induced Tumor Eradication and Inhibits Interleukin-7 Reduction in Non-Small Cell Lung Cancer. Biosci. Rep..

[18] Xiong F., Jiang M., Chen M., Wang X., Zhang S., Zhou J., Li K., Sheng Y., Yin L., Tang Y. (2017). Study on Inhibitory Effect of MaiMenDong Decoction and WeiJing Decoction Combination with Cisplatin on NCI-A549 Xenograft in Nude Mice and Its Mechanism. J. Cancer.

[19] Wang H., Lu C., Zhou H., Zhao X., Huang C., Cheng Z., Liu G., You X. (2025). Synergistic Effects of Dihydroartemisinin and Cisplatin on Inducing Ferroptosis in Gastric Cancer through GPX4 Inhibition. Gastric Cancer Off. J. Int. Gastric Cancer Assoc. Jpn. Gastric Cancer Assoc..

[20] Fu D., Wang C., Yu L., Yu R. (2021). Induction of Ferroptosis by ATF3 Elevation Alleviates Cisplatin Resistance in Gastric Cancer by Restraining Nrf2/Keap1/xCT Signaling. Cell. Mol. Biol. Lett..

[21] Wang F., Liu Y., Wang L., Bai Y. (2025). Ginkgetin Reverses Cisplatin Resistance in Cervical Cancer by Regulating the Nrf2/HO-1 Signaling Pathway to Induce Ferroptosis. Sci. Rep..

[22] Wu H., Zhang Z., Cao Y., Hu Y., Li Y., Zhang L., Cao X., Wen H., Zhang Y., Lv H. (2024). A Self-Amplifying ROS-Responsive Nanoplatform for Simultaneous Cuproptosis and Cancer Immunotherapy. Adv. Sci..

[23] Han L., Li L., Wu G. (2022). Induction of Ferroptosis by Carnosic Acid-Mediated Inactivation of Nrf2/HO-1 Potentiates Cisplatin Responsiveness in OSCC Cells. Mol. Cell. Probes.

[24] Lou J., Zhao L., Huang Z., Chen X., Xu J., Tai W., Tsim K., Chen Y., Xie T. (2021). Ginkgetin Derived from Ginkgo Biloba Leaves Enhances the Therapeutic Effect of Cisplatin via Ferroptosis-Mediated Disruption of the Nrf2/HO-1 Axis in EGFR Wild-Type Non-Small-Cell Lung Cancer. Phytomed. Int. J. Phytother. Phytopharm..

[25] Zhao K., Wen L. (2018). DMF Attenuates Cisplatin-Induced Kidney Injury via Activating Nrf2 Signaling Pathway and Inhibiting NF-kB Signaling Pathway. Eur. Rev. Med. Pharmacol. Sci..

[26] Kavian N., Mehlal S., Jeljeli M., Saidu N., Nicco C., Cerles O., Chouzenoux S., Cauvet A., Camus C., Ait-Djoudi M. (2018). The Nrf2-Antioxidant Response Element Signaling Pathway Controls Fibrosis and Autoimmunity in Scleroderma. Front. Immunol..

[27] Yang W., Wang Y., Zhang C., Huang Y., Yu J., Shi L., Zhang P., Yin Y., Li R., Tao K. (2022). Maresin1 Protect Against Ferroptosis-Induced Liver Injury Through ROS Inhibition and Nrf2/HO-1/GPX4 Activation. Front. Pharmacol..

[28] Hou C., Suo Y., Lv P., Yuan H., Zhao L., Wang Y., Zhang H., Sun J., Sun L., Lu W. (2025). Aristolochic Acids-Hijacked P53 Promotes Liver Cancer Cell Growth by Inhibiting Ferroptosis. Acta Pharmacol. Sin..

[29] Luo X., Linghu M., Zhou X., Ru Y., Huang Q., Liu D., Ji S., Ma Y., Luo Y., Huang Y. (2025). Merestinib inhibits cuproptosis by targeting NRF2 to alleviate acute liver injury. Free Radic. Biol. Med..

[30] Yin M., Palsson-McDermott E., Henry Ó., Ge Z., Toller-Kawahisa J., Min Y., McGettrick A., Gordon A., Heffernan S., Marrone L. (2025). Mitochondrial Glutathione Transporter SLC25A40 Regulates Macrophage Cytokine Production. Sci. Rep..

[31] Liu J., Tang H., Chen F., Li C., Xie Y., Kang R., Tang D. (2024). NFE2L2 and SLC25A39 Drive Cuproptosis Resistance through GSH Metabolism. Sci. Rep..

[32] Du M., Fu J., Zhang J., Zhu Z., Huang X., Tan W., Liu L., Huang Z., Liu X., Tan Q. (2024). CircSpna2 Attenuates Cuproptosis by Mediating Ubiquitin Ligase Keap1 to Regulate the Nrf2-Atp7b Signalling Axis in Depression after Traumatic Brain Injury in a Mouse Model. Clin. Transl. Med..

[33] Zhang M., Shi M., Yu Y., Ou R., Ge R., Duan P. (2024). Curcuminoid PBPD Induces Cuproptosis and Endoplasmic Reticulum Stress in Cervical Cancer via the Notch1/RBP-J/NRF2/FDX1 Pathway. Mol. Carcinog..

[34] Qiao L., Zhu G., Jiang T., Qian Y., Sun Q., Zhao G., Gao H., Li C. (2024). Self-Destructive Copper Carriers Induce Pyroptosis and Cuproptosis for Efficient Tumor Immunotherapy Against Dormant and Recurrent Tumors. Adv. Mater..

